# 3D Printing Improved Testicular Prostheses: Using Lattice Infill Structure to Modify Mechanical Properties

**DOI:** 10.3389/fsurg.2021.626143

**Published:** 2021-04-20

**Authors:** Jacob Skewes, Michael Y. Chen, David Forrestal, Nicholas J. Rukin, Maria A. Woodruff

**Affiliations:** ^1^Engineering Faculty, Queensland University of Technology, Brisbane, QLD, Australia; ^2^Herston Biofabrication Institute, Metro North Hospital and Health Service, Brisbane, QLD, Australia; ^3^Redcliffe Hospital, Metro North Hospital and Health Service, Brisbane, QLD, Australia; ^4^School of Medicine, University of Queensland, Brisbane, QLD, Australia

**Keywords:** 3D printing, testicular prosthesis, meta-materials, bio-fabrication, implants, soft prostheses, bio-materials

## Abstract

Patients often opt for implantation of testicular prostheses following orchidectomy for cancer or torsion. Recipients of testicular prostheses report issues regarding firmness, shape, size, and position, aspects of which relate to current limitations of silicone materials used and manufacturing methods for soft prostheses. We aim to create a 3D printable testicular prosthesis which mimics the natural shape and stiffness of a human testicle using a lattice infill structure. Porous testicular prostheses were engineered with relative densities from 0.1 to 0.9 using a repeating cubic unit cell lattice inside an anatomically accurate testicle 3D model. These models were printed using a multi-jetting process with an elastomeric material and compared with current market prostheses using shore hardness tests. Additionally, standard sized porous specimens were printed for compression testing to verify and match the stiffness to human testicle elastic modulus (E-modulus) values from literature. The resulting 3D printed testicular prosthesis of relative density between 0.3 and 0.4 successfully achieved a reduction of its bulk compressive E-modulus from 360 KPa to a human testicle at 28 Kpa. Additionally, this is the first study to quantitatively show that current commercial testicular prostheses are too firm compared to native tissue. 3D printing allows us to create metamaterials that match the properties of human tissue to create customisable patient specific prostheses. This method expands the use cases for existing biomaterials by tuning their properties and could be applied to other implants mimicking native tissues.

## Introduction

Testicle prostheses are offered to patients following orchidectomy for testicular tumors, loss after torsion, atrophy, and undescended testicles. These prostheses are made from silicone, like breast implants, either being fully solid, saline-filled, or silicone gel-filled. There are theoretical risks associated with silicone and liquid-filled implants, including connective tissue diseases, auto-immune disorders, and implant failure due to rupture. While these risks remain very low, there is a market push toward an alternative for silicone use in soft prosthetic implant design (1–3).

The main issues regarding testicular implants are related to the use and limitations of current materials and manufacturing methods. Recipients of testicle prostheses often describe them as being too firm, not the right size or shape, or positioned too high. These aspects can adversely affect physical exercise, sexual activity and confidence, leading to dissatisfaction and regretting the decision for accepting an implant. Sizes for testicular prostheses offered on the market are limited to small, medium, and large despite the individual nature of the human body (4–7). Additionally, they are designed to be sutured in place only in one position at the top of the prosthesis allowing them to freely rotate around that point inside the scrotum (8). The solution to these problems may lie with adopting new approaches to customized implant design using 3D scanning, modeling, and printing. Size, shape, and suture positioning could be adapted to an individual's needs using these technologies. However, a significant challenge for improving the firmness or “feel” of prosthetic testicles is the lack of medically approved bio-materials with appropriate properties.

To the best of our knowledge there is currently no study which quantifies and compares current market testicular prostheses regarding firmness, size and shape to verify patient complaints or provide a benchmark for creating an improved prosthesis. The use of 3D printing in individual testicle prosthesis design is limited and does not focus on developing prostheses with the intention to solve issues regarding firmness, soft tissue applications or using 3D printing to directly print an implant (9).

Consequently, it would be highly beneficial to create a system where already approved, well-established materials could be manufactured using innovative techniques to alter material properties. Doing so would allow a testicular prosthesis or other soft prostheses to have controllable properties that match natural tissue.

3D printing processes typically create components layer by layer from 3D computer models. Currently, 3D printing is the only method which can produce precisely controlled lattice filled structures. Research with lattice filled structures to create bio-mimetic properties has been extensively used with metals and primarily focused on mimicking bone and hard tissue properties (10–12). By creating a soft prosthesis using this method, we can overcome common problems described by patients, while avoiding the need for silicone gels or liquid infills to develop a safer, more natural-feeling product. In this study, we aim to prototype a 3D printed testicular prosthesis by engineering a lattice structure that exhibits the natural feel, size and shape of a real testicle.

**Figure 1:**
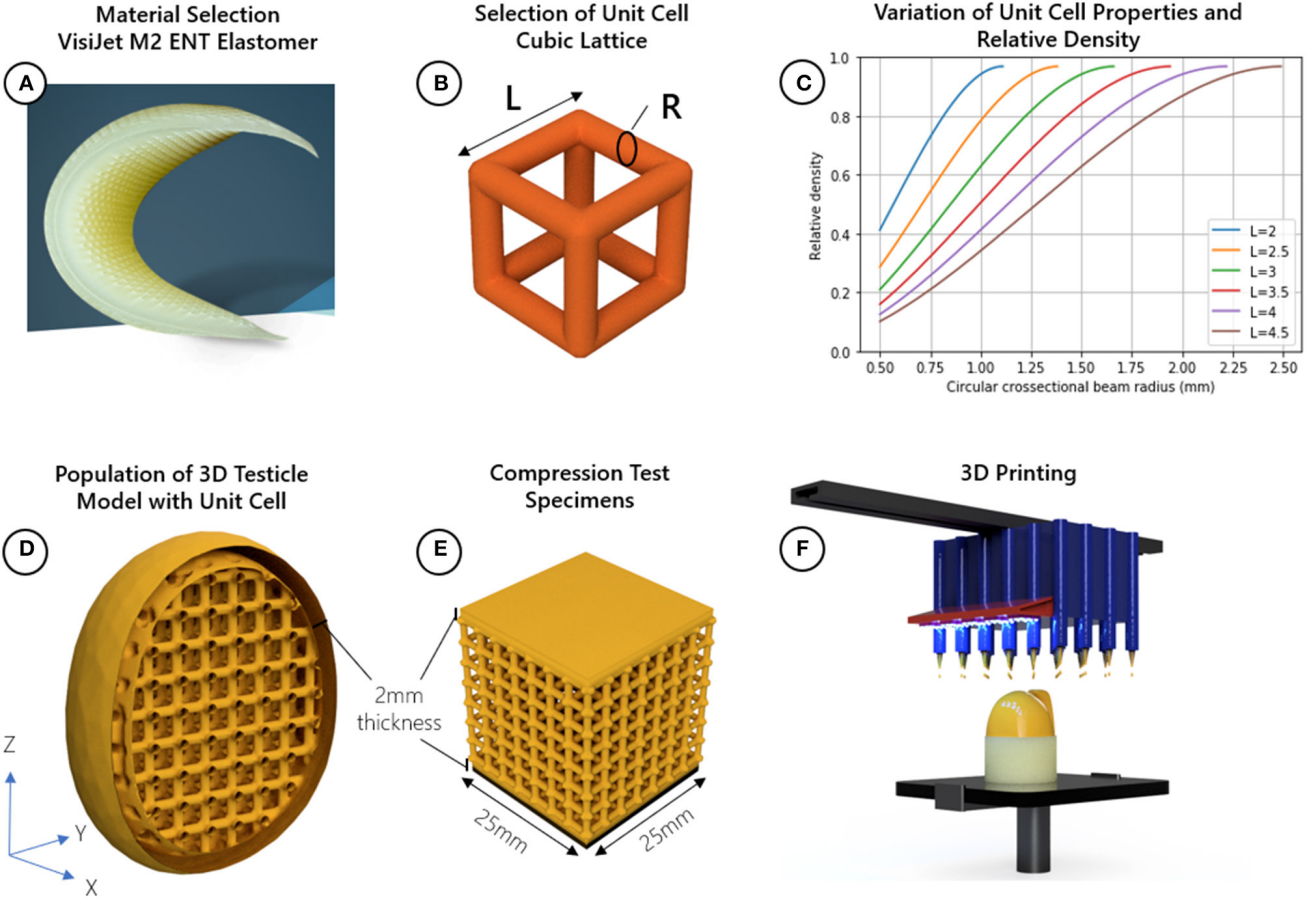
Method for designing testicular prostheses; **(A)** material selection, **(B)** selection of cubic lattice unit cell and identification of relevant design parameters related to relative density length (L) and radius (R), **(C)** Plot showing how the radius and length of the cubic unit cell beams can be adjusted to achieve a range of relative densities, choosing a radius, or length which is too small may not be manufacturable, while choosing values too large narrows the range of achievable relative densities, **(D)** populate 3D model of testicle with cubic unit cell, **(E)** create compression test specimens with uniform cross-sectional area (25 mm^2^), **(F)** 3D print the testicles and compression specimens using a Multi-Jet process.

A meta-material is a material engineered to have particular mechanical properties based on its sub-structure and not material composition. For example, two foams made from the same material can be either stiff or soft depending on their relative densities. However, while foam is made with randomized sub-structures, a meta-material is populated with a repeating shape called a unit cell. Properties such as the E-modulus (stiffness) and hardness can be finely tuned by manipulating the physical parameters of the unit cell. This approach was used to design a 3D printed testicular prosthesis with anatomical shape, size, and tuneable stiffness. Figure 1 outlines the method for designing the testicular prostheses.

## Materials and Methods

### Testicular Prosthesis Design

Testicle prostheses and standard sized samples were 3D printed using Visijet M2 ENT UV Curable Elastomeric material with a Projet MJP 2500 3D printer (3D Systems, Rock Hill, United States). The reported E-modulus (*E*_*s*_), strength (σ_*s*_), and density (ρ_*s*_) of this material was 0.27–0.43 MPa, 0.2–0.4 MPa, and 1.12g/cm^3^, respectively (13). The unit cell chosen to adjust the mechanical properties of the base material is the cubic lattice structure made of circular cross-sectional beams with a length (*l*) and radius (*r*). This unit cell was chosen because of its simplicity and known analytical relationships. The relative density can be adjusted using Equation (1) and the E-modulus (related to firmness, hardness and feel) can be adjusted using Equation (2).

(1) $$\begin{array}{rcl} \frac{\rho }{\rho_{s}} & = & 3\pi {\left(\frac{r}{l}\right)}^{2}-8\sqrt{2}{\left(\frac{r}{l}\right)}^{3} \end{array}$$

(2) $$\begin{array}{rcl} \frac{E}{E_{S}} & = & \frac{\pi r^{2}}{l^{2}} \end{array}$$

These equations are based on the applied mechanics Euler-Bernoulli beam theory which suggest that a minimum of six unit cells in the X, Y, and Z dimensions of a sample is needed for the analytical solutions to begin to converge to real world expectations (14, 15).

Testicles with relative densities 0.2, 0.3, 0.4, 0.5, 0.6, 0.7, 0.8, 0.9, and 1.0 (fully solid) using the cubic lattice structure were designed. The testicular prosthesis model and shape was adapted from the BodyParts3D database (16), a collection of anatomical 3D models of an adult human male. The dimensions of the testicle model used were 29, 31, and 38 mm in the X, Y, and Z directions, respectively. Considering the size of the model and to achieve the relative densities, the length of the unit cells were held constant at 3.5 mm while the radius of the beams were adjusted as depicted in Figure 1C. A wall thickness of 2 mm was chosen to provide the unit cells with a protective skin while minimizing any additional stiffness contribution to the prosthesis.

Compression tests with different shaped objects cannot be compared since the shape and cross-sectional area affects how much force is needed to displace a material. Additionally, shapes with non-uniform cross-sections cannot yield E-modulus values. Standard sized test samples with a uniform cross-section provide a way for determining the E-modulus of the lattice structures independent of its final shape. Thus, to verify the analytical models through compression testing, cube-shaped samples of uniform cross-sectional area (25 × 25 mm) using the same design parameters (unit cell size, shape, and top and bottom wall thickness) as the testicular prostheses were created using 3D printing (Figure 1E).

All 3D models were designed in 3D Sprint software package (3D Systems, Rock Hill, United States), a tool for manipulating parameters such as wall thickness, unit cell type, model size, and preparing the final models for 3D printing.

### Testicular Prosthesis Manufacturing and Post-processing

**Figure 2:**
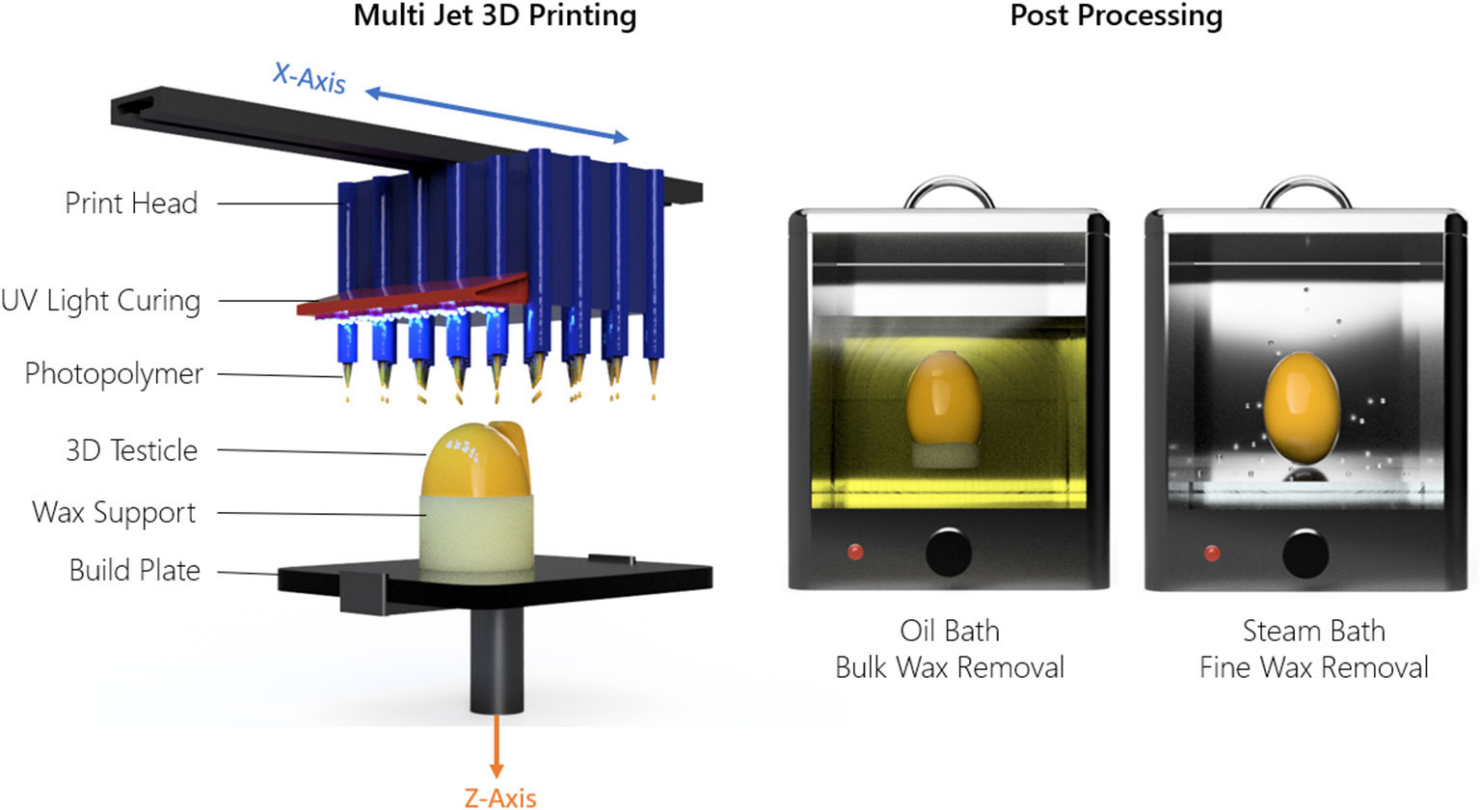
Material jetting technique and post-processing method used to 3D print the testicle prostheses.

The Projet MJP 2500 3D printer uses an inkjet printing head which dispenses droplets of a liquid photopolymer onto a build surface. The droplets are subsequently hardened by Ultra-Violet (UV) light. A wax support structure is simultaneously printed to fill any voids in the 3D model which requires removal post process in a hot water bath followed by an oil bath. This process is outlined in Figure 2. Using the 3D printer detailed here, the achievable X, Y, Z resolution is 1,600 × 900 × 90 DPI, with a recommended minimum feature size of 25 μ*m*. Based on the DPI, the resulting layer thickness in the Z direction was 32 μ*m*. The printer parameters used regarding speed, curing time, temperature, and layer thickness were set at factory default settings by 3D Systems and cannot be changed since these parameters are optimized specifically to the MJF 2500 print head and Visijet M2 ENT UV material.

### Compression Testing

Compression testing was conducted using an Instron Universal Testing System (Norwood, United States) with a 2 kN load cell compressing at 10 mm per min at a temperature of 25°C to 50% strain, along the Z axis of print direction for all samples. Cubic shaped samples of uniform cross-sectional area (Figure 1E) were compressed to verify the analytical model and determine the E-modulus (stiffness) of the bulk material and the relative densities. Values obtained from previously published studies using Real-Time Shear Wave Elastography to measure the E-modulus of male testicles were used to verify that the designed prostheses fell within the range of real testicular stiffness (28 ± 6 KPa) (17–19). For each relative density three samples were tested for statistical analysis.

### Shore Hardness Testing

Shore hardness is a measure of a materials resistance to indentation using a device with a spring-loaded indenter. Using a Shore OO Durometer device (Hildebrand, Zürich, Switzerland) we measured the hardness of the 3D printed prostheses and medical testicle prostheses, the gold standard comparison; Promedon (Endotherapeutics, Epping, Australia), Kiwee (Coloplast, Humlebaek, Denmark), and the Torosa (Coloplast, Humlebaek, Denmark). These prostheses were categorized into four areas (top, bottom, left and right) to assess any change in hardness due to features or shape differences of the prostheses. The hardness was measured in the center of those areas at room temperature following the method of the ASTM D2240 standards.

## Results

### 3D Printed Testicular Prostheses and Cubic Lattice Samples

**Figure 3:**
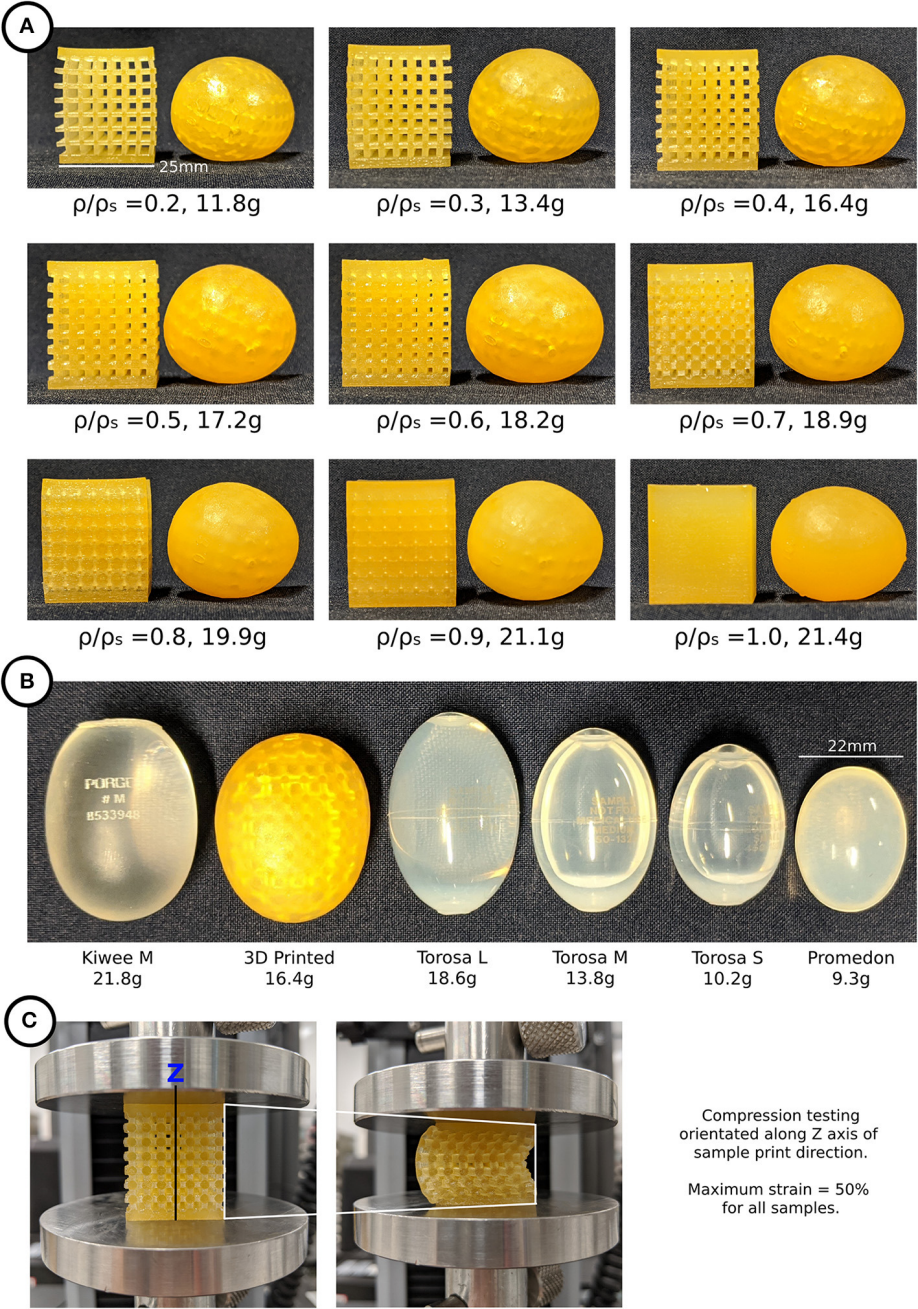
**(A)** 3D printed testicular prostheses and cubic lattice compression samples of relative density 0.2–1.0, **(B)** Medical prostheses Kiwee, Torosa, and Promedon (L, large; M, medium; S, small) next to a 3D printed testicular prosthesis, **(C)** Compression testing of cubic lattice samples showing deformation.

All 27 testicular prostheses and 27 cubic lattice samples, 9 of which are illustrated in Figure 3A, were printed together in 11.5 h. The average weight and material cost per prosthesis was 17 g and $16 AUD, respectively. A visual comparison of the market prostheses and a 3D printed testicular prosthesis with a relative density of 0.4 is shown in Figure 3B. The resulting testicular prostheses and samples appeared to have a smooth surface finish and layer lines often seen with 3D printed parts were not visible. Figure 3C captures the deformation of a 0.4 relative density cubic lattice sample under compression.

### Compression Testing Results

**Figure 4:**
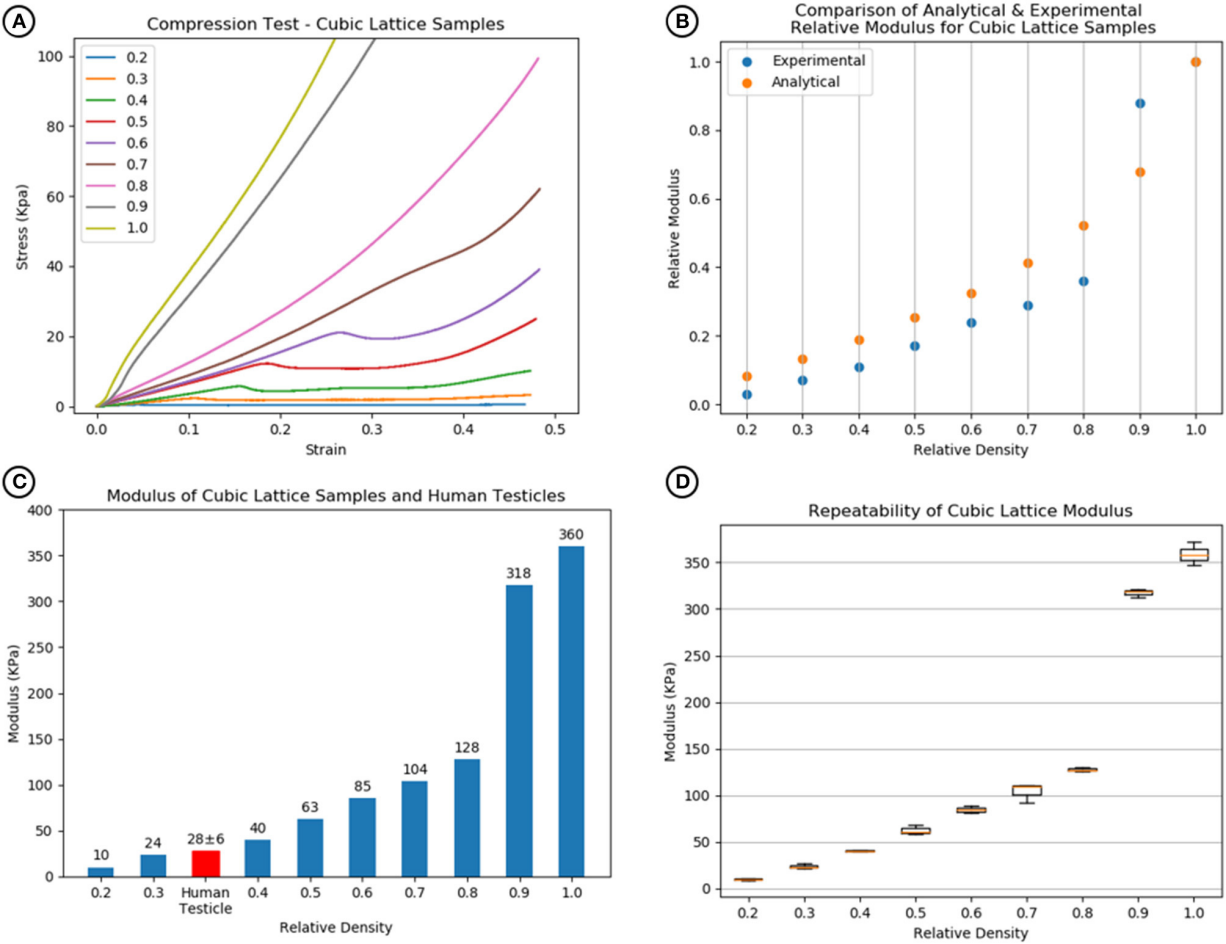
**(A)** Compression test results of cubic lattice specimens showing increase in stiffness with relative density. **(B)** Comparison of analytical [Equation (1) and (2)] and experimental relative modulus for cubic lattice specimens. **(C)** Relative densities of 0.3–0.4 match with human testicle stiffness values. **(D)** The repeatability of the modulus values across a sample size of 3 for each relative density.

Compression test results indicated that a 100% dense material with an E-modulus of 360 KPa is modifiable to a minimum of 10 KPa at 20% relative density. Figure 4B displays a comparison between the experimental and analytical results [from Equation (2)]. The averaged experimental results followed a similar trend; however, they are consistently lower than the analytical prediction apart from relative density 0.9. At this relative density, the pore sizes are too small to allow the wax support structure to be fully removed post printing, leaving excess solid wax behind and contributing to an increase in stiffness (Figure 4).

Comparison with human testicle E-modulus values demonstrated that the relative densities using this model required to match the feel of a testicle were between 0.3 and 0.4 as shown in Figure 4C. For each specimen, a boxplot in Figure 4D shows the repeatability of the cubic lattice E-modulus results for a sample size of 3.

### Hardness Testing Results

**Figure 5:**
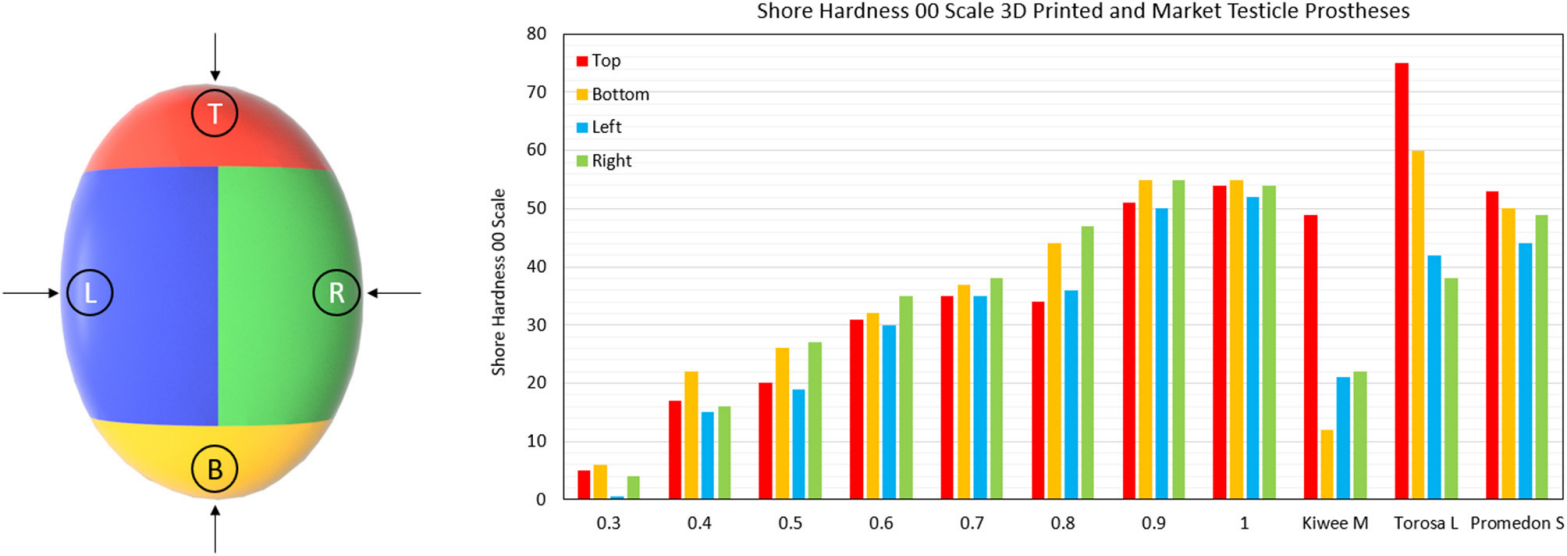
Shore Hardness OO scale results for 3D printed testicles and medical prostheses. Notably the Kiwee which is a silicone coated silicone gel filled prostheses shows appropriate hardness results (within ranges of relative density 0.3–0.4); however, Torosa and Promedon prostheses are relatively hard compared to natural tissue. Note that the Hardness results for Torosa small and medium sized prostheses are the same as the large size, as the hardness value is dominated by the material and not minor changes in size.

The results shown in Figure 5 show how the hardness of the 3D printed testicles correlates to its relative density while revealing the hardness of the market prostheses exceeded the hardness of samples with relative densities 0.3 and 0.4 which is the range we would expect the hardness of a human testicle to be (5–15). The top side of each market prosthesis was much harder than the left, right, and bottom positions because the suture holes located there were made from a harder material. Relative density of 0.2 was omitted from the results as these prostheses were too soft to obtain reliable values.

## Discussion

Using 3D printing we have demonstrated with a meta-material approach and a cubic lattice unit cell that we can manufacture a novel testicular prosthesis where the shape, size, and stiffness can be finely tuned to match a human testicle. With this approach, the need for silicone or liquid infills and thus risk of rupture is eliminated. Importantly, this approach expands the potential use cases of existing medically approved materials. This provides opportunity for a single base material to be used for a range of prosthetic implant products that vary in stiffness or other mechanical properties, with cost savings from economies of scale and reduced regulatory approval or material certification processes. These advantages and benefits are directly translatable to other areas of prostheses or silicone implant designs and to patients or clinicians looking for an improved individualized product.

To our knowledge, this is the first study to quantify and compare medical testicular prosthesis characteristics using a shore hardness test. The results shown here clearly indicate that the existing medical prostheses are relatively too firm. Particularly the top section where the suture position is located, consistent with patient complaints of products being too hard (4–6).

The best performing medical prostheses regarding hardness was the Kiwee, with results in-between relative densities 0.3 and 0.4 (the ideal case). However, in October 2017 Coloplast withdrew the Kiwee prosthesis from the market due to “particles found on the surface of some implants” (20). Being the only silicone gel filled testicle prostheses, the removal of the Kiwee from the market leaves only two options for patients at our hospital; the Torosa (saline filled) and Promedon (solid silicone).

Hardness tests provide a useful way to determine the basic feel of a prostheses. For 3D printing prostheses, hardness can be theoretically tuneable as there is a direct correlation between the hardness results and E-modulus values; as the relative densities increase, so does the hardness value.

The cubic unit cell used in this paper was a good starting point for prototyping a 3D printed testicular prosthesis with tuneable stiffness. However, the benefits of 3D printing can allow for more complex unit cell geometries, gradients of relative density, and wall thicknesses without compromising cost or the manufacturing process. A human testicle is made of several substructures, each with unique mechanical properties. These substructures could be mimicked in a more complex prosthesis design. For example, the epididymis could also be modeled into the prosthesis with its own firmness, as well as surgically functional aspects such as suture positions.

Such graded designed metamaterial approaches and 3D printing are beginning to be used to solve problems with or improve upon other medical device products. This includes applications for orthopedic implants where bone substructures including cortical and trabecular bone have been 3D printed with titanium in a graded lattice structure to match the anisotropic mechanical properties of bone (11, 21). These concepts could be further applied here to improve upon the testicle prosthesis design.

This technology could potentially have applications in other implants that seek to mimic the feeling of native tissue. For example, silicone breast implants have rupture rates as high as 10% at 10 years after implantation (22). Our method could create realistic feeling implants that have lower risk of rupture or shrinkage due to the lattice infill. Additionally, nose and ear prostheses or functional medical devices and training models which require hardness in some areas and softness in others could be achieved, owing to the versatility of printing differing structures in one print.

Different unit cells such as Triply Periodic Minimal Surfaces like the Gyroid, or Schwarz P surfaces, or other beam-based unit cells like the diamond can achieve different ranges of E-modulus for the same relative densities as the cubic unit cell (23). For example, the diamond structure can theoretically achieve a relative modulus of 0.05 with a relative density of 50%. Compared with the cubic which can achieve a relative modulus of 0.25 at relative density 50%. This means that by selecting the appropriate unit cell shape, a material which has a higher E-modulus could be used to achieve the same result, further expanding the use of existing materials.

### Limitations of Current Design

A limitation of this study is that the 3D printing material used is not approved for implantation. There is a lack of available and directly 3D printable soft polymer materials on the market that can be utilized to make patient specific and life-like implants, which provides a great opportunity for the 3D printing industry to develop compatible materials. Additionally, no fatigue tests have been conducted to determine if the cubic lattice structures withstand repeated loading. This is recommended for future work on implantable materials prior to clinical trials.

Although this study was not performed with an implantable material, the concept could be applied to an implantable material with a similar bulk E-modulus range and a variety of 3D printing methods including Selective Laser Sintering (SLS) or Fused Deposition Modeling (FDM). For example, materials such as Thermoplastic Polyurethane (TPU) which are approved for implantation such as Lubrizol Carbothane™ TPU (24) or Biomerics Quadrathane™ TPU (25) could be 3D printed using selective laser sintering (SLS) or fused deposition modeling (FDM) processes. These materials are used with injection molding systems and come in pelletised form. Therefore, they would need to be further processed into a powder or filament to be 3D printed. There are many of these TPU materials with stiffness ranges between 2.2 and 5.5 MPa. While stiffer than the material used here, using the concept in this paper with a diamond unit cell and a TPU with 2.4 MPa, a relative density of 0.25–0.35 would be recommended to match the feel of a human testicle.

## Conclusions

We have demonstrated how 3D printing can be used to create a meta-material lattice structure for realistic feeling testicular prosthesis prototypes. These prototypes address the most common complaints of patients including unnatural size and shape and discomfort. This same technology could be used with various elastomeric materials and simulate the characteristics of a variety of native tissues. These developments will contribute to a future of individualized patient implants and prostheses through complete customization of shape, size and mechanical properties matching of human tissue.

## Data Availability Statement

The original contributions presented in the study are included in the article/supplementary material, further inquiries can be directed to the corresponding authors.

## Author Contributions

All authors listed have made a substantial, direct and intellectual contribution to the work, and approved it for publication.

## Conflict of Interest

The authors declare that the research was conducted in the absence of any commercial or financial relationships that could be construed as a potential conflict of interest.
